# American Medical Association, Section on Stomtatology, Atlantic City, June 7 to 10, 1904

**Published:** 1904-08

**Authors:** 


### AMERICAN MEDICAL ASSOCIATION, SECTION ON STOMTATOLOGY, ATLANTIC CITY, JUNE 7 TO 10, 1904.

### DISCUSSION ON PAPERS IN SYMPOSIUM ON DENTAL EDUCATION BY DRS. EAMES, CHITTENDEN, BALDWIN, AND MARSHALL.

*Dr. Eugene S. Talbot,* Chicago.—Anticipating that something might be said in the Chairman's Address in regard to the length of term in the dental colleges, and the position that would be taken at the meeting in Washington, I wrote letters to Professor Eliot of Harvard and to President Harper of the University of Chicago, asking them to prepare papers for this section. President Harper was unable to comply with my request, but I have a letter from Professor Eliot, which I will read at this time:

"Dear Sir,—I shall not be able to attend the meeting of the Section on Stomatology of the American Medical Association at Atlantic City, June 7 to 10, 1904. The proper person to state the position of Harvard in regard to the future policy of its Dental School is the dean of that school, Dr. Eugene H. Smith, 283 Dartmouth Street, Boston.

"The policy of the Harvard School is not commercial in the least. It is steadily endeavoring to raise the standard of dental education, and has in a good degree succeeded. Just now it is trying to raise the standard of the admission examinations to the school, and is finding that a difficult task. It can not add, at the same time, to the length of its own course. Moreover, the school has been struggling for several years to maintain a close connection with the Harvard Medical School. The task has proved difficult because of the rapid improvement of the Medical School consequent on its admitting none but the persons who already hold a degree in Arts or Science. The Dental School must accomplish both the tasks to which it has set itself before it can lengthen its course to four years. The Section on Stomatology may rest assured that the Harvard Dental School will continue to do what it always has been doing—namely, contribute to the improvement of the preparatory course for the dental profession.

"Very truly yours,

"CHARLES W. ELIOT."

I am sorry to say that Dr. Smith can not be present, but Dr. Briggs will give us some idea of what the school will do.

*Dr. E. C. Briggs,* Harvard Medical School.—I am sorry that Dr. Smith can not be here to give you the best idea, and as it was only a few days ago that he told me he could not be present, I have not been able to make any real preparation or collect any special data. I can, however, point out one or two things and state a few facts which I hope will impress on your minds the idea that Harvard is not blocking in any way dental education. In fact, it seems ludicrous that others should have placed her in such a position, when she has always been struggling quite the other way, and, I may say, struggling against her own commercial interests. We have decimated our classes time and again by the advances we have made over other schools.

The question is, What elevates the profession? We speak about the progress we have made, but we lose sight of what really elevates the profession as a whole. There are plenty of men in the profession to-day who have elevated themselves as high as any one could wish, who have not had any particular preliminary training. They have had the genius and have made the geniuses, but that does not raise the profession as a whole. That is the feeling that we have at Harvard. To set the profession right before the world, attention must be given to the character of the men and their scholarly attainments, and that is the reason we have paid so much more attention to preliminary requirements than we have to the length of the course. The length of the course is a little misleading. It is four years of seven months; Harvard already has three years of nine months. We are only one month short. With that, and with our entrance requirements, we do not feel that we could wipe out our school altogether. We have about cut it in half now, and what better evidence can you have that we are not commercial? If we were commercial we would take in some of the men who come from other colleges who write to know what their position will be on coming into our school. Not one has accepted, because we say, we will give you time allowance, but you must pass every examination before you can graduate. That is not what they want, and they do not come. For the post-graduate our terms would be entirely different. That does away with the suggestion that our line of thought is due to any commercialism. I wish that you would send, as we have done, for purposes of comparison, to the different colleges, and see what their examination papers are and compare them with ours. Not one man out of ten with a high-school education can come into our school. He must pass the examination with ability to go into college—not twenty-four points, but as many points as he goes. He has got to pass just as good an examination as if to enter Harvard or Yale—I speak of them because they are nearer. So it seems to me that Harvard has been put in an unfortunate light, diametrically opposite to what she really is at heart. The time that a man is in a professional school is of great value to him; we appreciate that as much as anybody else—I do not believe ten years would be too much, if you wish to reach the highest ambition of life. The idea is, that having provided a certain amount of good training, which we think we do in three years, the next step is to have the man who comes into the school one who is already a scholar and fitted to take up any of the liberal professions. You and I are helped just as much by that uplift. We can not stand back and say we did pretty well and that we only had "reading, writing, and arithmetic." If the standard is raised by making it necessary to have something more than that, you will get professional standing. The profession will not make its highest success until the men who come into it are men of education and scholarly attainments.

*Dr. Williams,* Boston.—One of the things which I thought I would object to in teaching in Harvard was that I would have to teach with men who were not prepared to learn. I am glad to know that the standard of the examination has been advanced so that the students are better able to learn. The preliminary preparation is necessary in order to know how to learn to the best advantage.

*Dr. James Truman,* Philadelphia.—I feel in a somewhat embarrassing position, coming down here as if I were inspired to attack Harvard. That is not my purpose. So far as I am concerned, I want to advance dental education along the best line possible. In 1884 we organized the Association of Faculties, and for twenty years we have worked steadily to bring up the standard of education to four years. At Asheville last year, when we supposed we had established that, without any prospect of its being changed, we received a blow from the very highest educational centre in this country,—Harvard. It requested that it should be given the privilege of three years, because it intended to advance the preliminary standard to the A.B. degree eventually, and for that reason and for the higher standard which it claims at present, it believes that it had a right to a three years' course. I was chairman of the committee which took that matter in charge, and we could not, under the circumstances, agree to the proposition, because other universities in this country had practically the same standard and the same length of time. Now the action of Harvard on that occasion was an encouragement to the commercial schools. I know very well that Harvard has never been a commercial school. No one can charge Harvard with that. It has always been exactly the contrary, and that is why I was astounded at that time at its action. After they left the Association of Faculties we heard that we were to be met, first at Buffalo and in a day or two at Washington, with the commercial idea of going back to three years because the schools could not maintain themselves. Now, Harvard may not have meant this. Harvard did not mean it, but at the same time that influence has been an injury to general dental education in this country, and I do not see how Harvard can explain it.

We have had this afternoon a good deal that is of value in these papers, and, as a rule, I am in unity with them, especially with the paper of our chairman. I agree with the proposition that the higher in a general way you can educate a man the better, but you must have that education properly arranged in order to be of value. The idea to me is pre-eminently absurd to suppose that because a man has an A.B. degree that he is better qualified thereby to become a dentist. It is a fallacy. The high-school degree is equally fallacious. What we need in dentistry is an education that will fit a man to become a dentist. To accomplish this he should come up from the mechanical school, through the higher schools, and then reach a point where he can be of value to himself and the community at large. There is probably not a man here in this room who ever had more than two years of four or five months' study in the professional schools. I do not believe that we can make dentists out of all men, whether they have the A.B. degree (and we have a number of them) or whether they have the high-school degree; that is not the point. We must go deeper if we expect to have symmetry in our educational effort. My own idea has been that the poor dentists we have turned out have, as a rule, been men of higher education—not the fault of the higher education, but the fault of a lack of proper symmetry in that education. Let me illustrate: I had two boys to educate. One was my own son, the other a nephew. My own son I insisted should take a six years' course to acquire a proper classical education. After several years' preparatory training in a German gymnasium, he graduated with the highest honors of the university and with nothing in anything else. The other boy was mechanical. I insisted on his going through the manual training and from the manual training school to the university, and from the university to a post-graduate department in one of our large manufacturing establishments, making nearly eight years in his particular direction, and at the present time, at thirty years of age, he is superintendent of motive power in one of our largest establishments in Philadelphia. My own son became a teacher. That is what I mean by symmetry in education; training the men from the very beginning so that they can practise whatever they expect to adopt as their calling, and not supposing that because a man has an A.B. degree or any other degree of that character, that he can enter a dental school and become a dentist. This very day I have before me numbers in our own university who are in degree unfit to practise dentistry, and it is a source of tribulation to me that I have to meet that sort of thing. They have not been properly trained from the beginning. They have not the manipulative ability that ought to come with good practice, and they will never get it, because they have reached an age when it is impossible that that shall be perfected in the individual. If we are to advance education we must look at it intelligently. Let us come down to practical matters. Let us take our experience; the experience with me covers nearly half a century. In the old laws regulating mechanical work in my own State, young men had to go into apprenticeship, working for four or five years until they reached their majority at twenty-one, before they could practise any of the mechanical trades. Then they were skilled in their work. Now we are trying to turn out men in three years in one of the most complex mechanical professions that I know anything about in this world, that of dentistry. We would turn them out with a fundamental knowledge of anatomy, bacteriology, histology, etc., and the mechanical training almost entirely ignored. Is that what we are here for? Are we here for that purpose alone? If we are, then I am not a dentist.

*Dr. Williams,* Boston.—Dr. Truman is right in his views regarding the manipulative qualifications of a dentist. It is necessary that they should be trained properly and early. I have known some of the best manipulators to be the greatest failures in their operations from not knowing when and how to exercise their manipulative skill. I have known of one or two instances of fatal results, from very high skill misplaced.

